# Fine Mapping of Posttranslational Modifications of the Linker Histone H1 from *Drosophila melanogaster*

**DOI:** 10.1371/journal.pone.0001553

**Published:** 2008-02-06

**Authors:** Ana Villar-Garea, Axel Imhof

**Affiliations:** Munich Center for Integrated Protein Science CIPSM, Histone Modifications Group, Adolf-Butenandt Institute, Ludwig-Maximilians University of Munich, Munich, Germany; Texas Tech University Health Sciences Center, United States of America

## Abstract

The linker histone H1 binds to the DNA in between adjacent nucleosomes and contributes to chromatin organization and transcriptional control. It is known that H1 carries diverse posttranslational modifications (PTMs), including phosphorylation, lysine methylation and ADP-ribosylation. Their biological functions, however, remain largely unclear. This is in part due to the fact that most of the studies have been performed in organisms that have several H1 variants, which complicates the analyses. We have chosen *Drosophila melanogaster*, a model organism, which has a single H1 variant, to approach the study of the role of H1 PTMs during embryonic development. Mass spectrometry mapping of the entire sequence of the protein showed phosphorylation only in the ten N-terminal amino acids, mostly at S10. For the first time, changes in the PTMs of a linker H1 during the development of a multicellular organism are reported. The abundance of H1 monophosphorylated at S10 decreases as the embryos age, which suggests that this PTM is related to cell cycle progression and/or cell differentiation. Additionally, we have found a polymorphism in the protein sequence that can be mistaken with lysine methylation if the analysis is not rigorous.

## Introduction

Histone H1 is a linker histone, as it binds to the DNA that resides between two adjacent nucleosome particles (for review see [1]–[4]). The binding of H1 to nucleosomes is involved in the stabilization of the 30 nm chromatin fiber [5], [6], affects the structure and positioning of nucleosomes [7], [8] and influences the accessibility of nucleosomes to other proteins [9], [10]. As result, H1 contributes to chromatin organization, transcriptional control and is a key factor of cellular differentiation during early embryonic development [11].

Histone H1 consists of a conserved globular domain, which is sufficient for the binding of H1 to nucleosomes [12]–[14], and highly divergent N- and C-terminal extensions (tails), which are rich in lysine, alanine and proline [15]. The tails play important roles in the H1 induced chromatin condensation [16] and the binding to specific *loci* [17].

It is known that H1 from various organisms is posttranslationally modified at several positions (PTMs), including phosphorylation [18]–[22] and poly(ADP-ribosylation) [23]. More recently, some mammalian isoforms have been shown to be acetylated, methylated and formylated at lysine residues [19], [24]–[27]. The only modification that has so far been associated with particular cellular functions is the phosphorylation of serines [28]–[30] and to a lesser degree the methylation of lysines [24], [25]. However, many studies addressing the biological relevance of the PTMs have been performed in organisms that have more than one H1 variant, which complicates the design and the interpretation of the experiments. In order to study the linker histone function during early murine embryonic development, three isoforms had to be knocked out due to the high level of redundancy [5], [31]. Moreover, the amino acid composition of H1, in particular the abundance of lysine residues, challenges the analysis of the protein with techniques using specific proteases like mass spectrometry.

In order to better study the importance as well as the evolutionary conservation of H1 PTMs, we decided to analyze H1 from *Drosophila melanogaster* embryos by mass spectrometry. This model organism has several advantages with regards to the analysis of H1 modifications. It has a single variant of H1, the embryonic development is well characterized and it is possible to collect a sufficient amount of embryos in a relatively easy way. In *Drosophila*, H1 is first detected at the cycle 10, after zygotic transcription starts [32], when it replaces the HMG-D protein that compensates for the lack of H1 during early cleavage states [33], [34]. Previous studies have shown that H1 in *D. melanogaster* is phosphorylated [35] in its N-terminal region [36], however, the exact position of the phosphorylated sites has not yet been described. Moreover, it has not been determined if H1 in this organism carries other PTMs besides phosphorylation. In contrast to other techniques, mass spectrometry neither requires the assumption of the existence or absence of a certain modification, like the antibody-based techniques, nor the presence of a certain number of electric charges on it, like the electrophoresis.

### Analysis of Histone H1 in 0–12 h embryos

To develop a robust protocol that allows a parallel analysis of several samples of H1 from Drosophila embryos from different stages, we initially used embryos collected between 0 to 12 h after egg laying (a.e.l.). One of the major inconveniences when working with early embryos is the enormous amount of yolk present in the protein extracts. To circumvent this problem, nuclei are separated from the bulk of yolk proteins and subsequently extracted with perchloric acid. The resulting extract is highly enriched in histone H1 and HMG-D. After dialysis and freeze-drying, histone H1 can be easily purified by RP-HPLC. The use of HPLC for protein separation has a clear advantage over the use of SDS-PAGE: the isolated proteins remain in solution, and many proteases employed for MS analysis do not cleave efficiently when the substrate is embedded in a gel piece.

**Figure 1:**
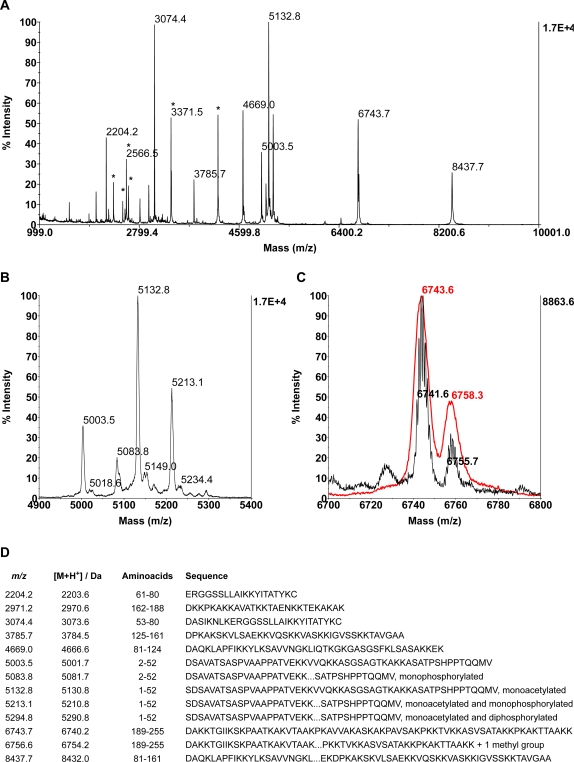
MALDI-TOF analysis of H1 from 0–12 h embryos after Asp-N digestion. H1 from 0–12 h embryos was purified and digested with Asp-N. Digestion mixtures were desalted and analyzed by MALDI-TOF mass spectrometry in positive, linear mode. A) A typical spectrum has signals corresponding to all the expected peptides. * labels the signals corresponding to [M+2H]^2+^. B and C) Zooms of the spectrum shown in A encompassing the two regions where signals corresponding to modified peptides are found. B) Peaks of the N-terminus of the protein (5003.5 and 5132.8) and its phosphorylated forms (5083.8, 5213.1, 5294.8). C) C-terminus of the protein (6743.7) and its presumptive methylated form (6756.6). Red: MALDI-TOF, linear positive mode; black: MALDI-TOF, reflector positive mode. D) Assignment of the peaks in A-C. *m/z*: experimental *m/z* values, [M+H]^+^: expected *m/z* values (accession number P02255), Amino acids: amino acids contained in the peptide, Sequence: aminoacid sequence of the corresponding peptide and indication of the presence of PTMs. Note that the aminoacids position are referred to the mature protein, without the first methionine.

Purified H1 was digested with the endoprotease AspN, which hydrolyzes the peptide bond N-terminal of aspartic acid, and MALDI-TOF spectra were acquired in the linear, positive mode. As shown in Fig. 1, the resulting peptide mixture covers the entire sequence of the protein. Only the peaks corresponding to the N- and the C-terminus of H1 in the spectrum show additional signals that could be explained by PTMs in H1.

In the N-terminus (Fig. 1B), we detect the expected peak for the peptide 2–52 and an additional signal matching with the monophosphorylated form of the same peptide. Due to a missed cleavage of the bond between S1 and D2, peaks corresponding to the acetylated peptide 1–52 and it's mono- and diphosphorylated forms are also detected. Given the low resolution of the spectra in the linear mode and the proximity of the expected signal for diphosphorylated 2–52 (5163.8) to other signals (sodium salt of monoacetylated 1–52 at 5154.8 and a neutral loss of a methylsulfoxide from the the oxidized 1–52p at 5272.7), the existence of diphosphorylated 2–52 cannot be determined with AspN digestion. We tried to acquire the spectra of these digests on the reflector mode, which has higher resolution, however the phosphate groups were unstable in the conditions of the measurement and we could only detect signals corresponding to unmodified peptides or to diverse neutral losses (data not shown).

Because the acetylation can only be seen in the peptide 1–52 and not in 2–52, we conclude that H1 is acetylated at the amino terminus. This conclusion is also supported by digestion with other proteases (trypsine, Glu-C) and by the MS/MS analysis of peptides carrying the N-terminus.

The peak corresponding to a C-terminal peptide (aa 189–255) shows only one additional mass at a m/z value of 6758.3 (Fig. 1C). The resolution of the spectrometer in the linear mode (red trace in Fig. 1C) did not allow us to determine if the second peak differs from 189–255 by 16 (oxidation) or 14 units (one methyl group). However, when a spectrum of the same sample was recorded in the reflector mode (Fig. 1C, black trace), the higher resolution allowed us to determine the difference as being 14.1 amu and could potentially be the result of a monomethylation within the peptide 189–255.

### A polymorphism in the C-terminus of H1 can be mistaken for a monomethylation

In order to further study the potential methylation, we decided to map the amino acid position(s) in the C-terminus of H1 at which the mass shift is observed. However, the composition of the peptide (22 lysine, 18 alanine, 7 threonine, 6 valine, 5 serine, 5 proline, 2 isoleucine, 1 glycine and 1 aspartic acid residue) and its size (6.7 kDa), made it very difficult to map the site of modification using conventional proteases or tandem mass spectrometry.

Therefore we decided to purify the peptide by RP-HPLC and subsequently perform a limited carboxypeptidase Y digestion on the purified peptides. The reaction products were then analyzed using MALDI-TOF MS. Carboxypeptidase Y removes the C-terminal amino acid of a peptide in a processive manner thereby resulting in a series of peaks that differ by the mass of one aminoacid. However, as the reaction rate is highly dependent on the nature of the removed amino acid, the resulting ladder is not continuous and only the peptides that contain an amino acid at the C-terminus, which is slowly cleaved by carboxypeptidase Y, can be seen.

**Figure 2:**
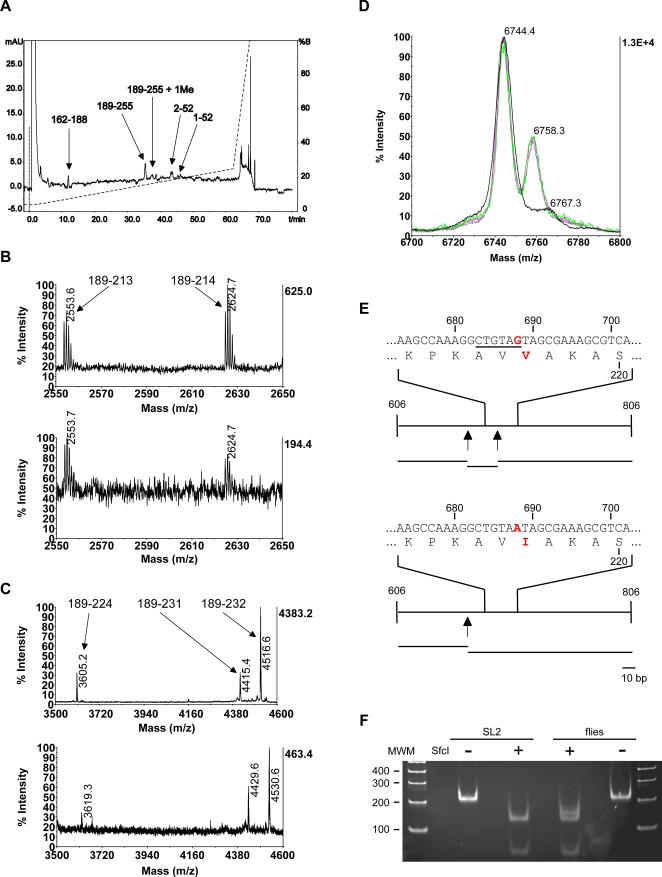
A polymorphism in the protein can be misinterpreted for a methylation in the C-terminus. A) Separation of the peptides produced by AspN digestion of H1 from 0–12 h embryos over a C18 column. Continous line: absorbance at 214 nm; dashed line: eluent composition expressed as percentage of eluent B. B and C) The purified peptides 189–255 and its methylated form were partially digested with carboxypeptidase Y. The digestion mixtures were desalted and analysed by MALDI-TOF mass spectrometry in the linear, reflector mode. Zooms are shown. In both panels, the upper spectrum corresponds to the unmodified form and the lower, to the modified one. The results indicate that the methylated residue is in the stretch 215–224. B) Region displaying the largest peptides where identical mass in both samples is detected. C) Region displaying the smallest peptides where a mass difference between both samples is detected. For the region 215–224 no signals are present in the spectra. D) H1 was isolated from different sources, digested with Asp-N, desalted and analysed by MALDI-TOF mass spectrometry in the linear, positive mode. Zoom of the region containing 189–255 is shown. Magenta: 2–3 h embryos, green: 12–15 h embryos, grey: 0–12 h embryos, black SL2 cells. The proportion unmethylated (6744.4)/methylated (6758.3) is identical in all the embryonic samples, whereas no methylated species can be detected in the SL2 cells. The peak with a m/z value of 6767.3 corresponds to a sodium adduct of the unmethylated form. E) The existence of the polymorphism V217I could explain our observations. Upper panel: a stretch of the DNA sequence of a H1 gene coding for the Val allele is shown (accession number: NM_165380). The numbers indicate the base position with respect to the transcription start. A SfcI restriction site is underlined. The corresponding translated sequence is shown underneath. The numbers indicate the aminoacid position respect to the mature protein. The long lines represent the fragment of DNA sequence amplified by PCR, the arrows, the SfcI restriction sites and the short lines at the bottom, the expected fragments after SfcI digestion. The lower panel represents the same but for a H1 gene coding for the Ile allele (for instance, accession number: NM_1032208). The shift 688 G->A results in the lost of a SfcI restriction site. F) The polymorphism Val217Ile is present in the fly population. A stretch of the coding sequence of H1 (606–806) was amplified from fly and SL2 cells genomic DNA. The products were digested with SfcI and the resulting fragments were analysed by polyacrylamide gel electrophoresis. As control, parallel incubations with no enzyme were performed. In the sample from SL2 cells, only the expected bands for the allele Val are observed (60 and 123 bp, the 18 bp bands ran out of the gel), whereas in the sample from the flies, the pattern fits with the existence of both the Val (60 and 123 bp) and the Ile (60 and 141 bp) alleles.

In our conditions, the peptides corresponding to the peptide 189–255 could be separated from its potentially methylated form by RP-HPLC (Fig. 2A). The carboxypeptidase Y digestions performed in parallel indicate that the modified amino acid resides between the residues 215 and 224, as the signals corresponding to 189–214 and smaller have identical m/z values, whereas the peaks assigned to 189–224 and longer show a mass shift of 14 amu.

We next wondered whether the ratio of the wild-type peptide and the shifted one change during development. In order to do this, we isolated H1 from embryos at different developmental stages (2–3 h, 3–6 h, 6–9 h, 9–12 h and 12–15h a.e.l.). A quantitative comparison of the relative intensities of the two peaks did not change during embryonic development (Fig. 2D). Therefore we concluded that the potential methylation is not regulated during development. In order to have an independent source of H1 molecules, we also extracted H1 from SL2 cells (cell line of embryonic origin) and analyzed the C-terminal peptides. In contrast to our expectations, H1 did not seem to be methylated at the C-terminus (Fig. 2D). As we also failed to observe a C-terminal methylation in H1 isolated from embryos derived from a different fly strain we wondered if there are alternative explanations for the observed mass shift.

One possible explanation could be the existence of different isoforms that differ by the mass of one methyl group. Indeed, several polymorphisms in the sequence of *D. melanogaster* H1 are recorded in the DNA and protein databases. One of them, V217I, could explain our observations. This polymorphism corresponds in the DNA sequence with G688A and results in the loss of a SfcI restriction site (Fig. 2E). To determine if this polymorphism is present in our fly population, a 201 bp fragment from H1 coding sequence encompassing the position 688 was amplified and digested with SfcI. DNA from SL2 cells was employed as control. The analysis of the resulting fragments by polyacrylamide gel electrophoresis is shown in Fig. 2F. In the sample from SL2 cells only the products of the digestion of the Val allele are detected (fragments of 60 bp and 123 bp). In contrast, in the sample from the embryos, digestion products of both alleles (fragments of 60 bp and 123 bp for the Val allele, fragments of 60 bp and 141 bp for the Ile allele) are present. Therefore, the signal with a m/z value of 6758 does not correspond to 189–255 with a methylated lysine residue, but to 189–255 with Val 217 replaced by Ile.

### Analysis of H1 phosphorylation in 0–12 h embryos

**Figure 3:**
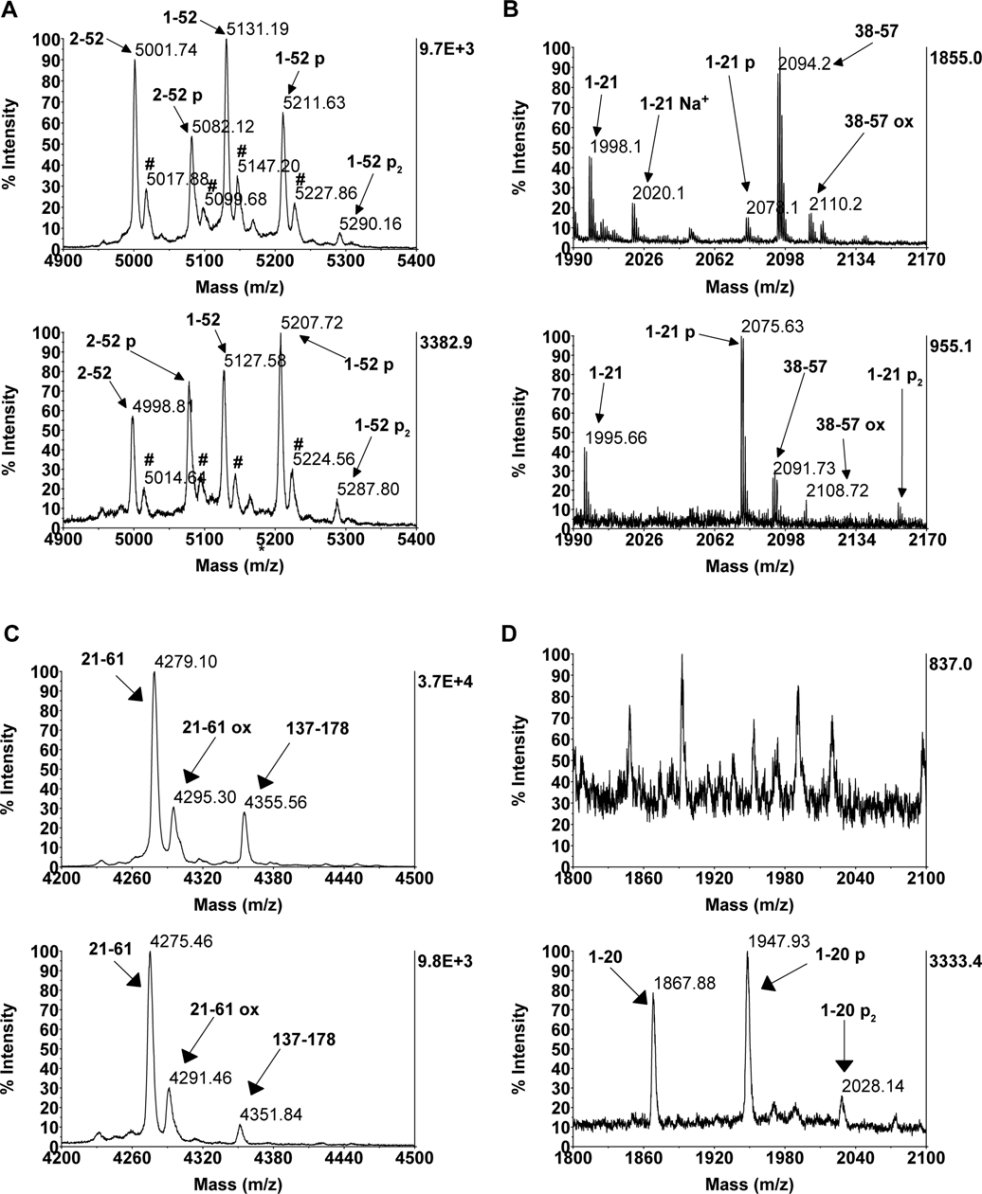
Phosphorylation in 0–12 h embryos is located in the N-terminal 20 aminoacids. H1 from 0–12 h embryos was isolated and digested with AspN (A), trypsin (B) or Glu-C (C, D). Digests were desalted and MALDI-TOF spectra were acquired in the linear (A, C, D) or reflector (B) modes. For each sample, spectra in the positive (upper spectrum on each panel) and negative (lower spectrum on each panel) were recorded. Zooms of the interesting regions are shown. A) In the negative mode, no more phosphorylation states for 1–52 mode are detected, respect to the positive mode. B) After digestion with trypsin, mono- and diphosphorylation are detected in 1–21 and no modification (apart from oxidation) is present in 38–57. C, D) After digestion with Glu-C, no PTM is detected for 21–61 (only oxidation, C), however 1–20 appears clearly un-, mono and diphosphorylated (D).

As mentioned above, histone H1 in 0–12 h embryos is phosphorylated at the N-terminus. In order to enhance the detection of putative phosphorylated species, MALDI-TOF spectra in the negative, linear mode were acquired from H1 after digestion with AspN. However, even under conditions where phosphopeptides are preferentially ionized not more than 2 phosphate groups can be detected in the peptide 1–52. This is also the only region of the protein appears to be stably phosphorylated (Fig. 1A and Fig. 3A).

To narrow down the number of possible modified sites, H1 was digested with other proteases and the resulting digestion products were analyzed by MALDI-TOF in positive and negative mode. Trypsin digests show that a significant proportion of the peptide 1–21 is mono- and diphosphorylated, whereas no modification apart from oxidation is observed in 38–57 (Fig. 3B). Glu-C digestion confirms that no phosphorylation is detected outside 1–20 (Fig. 3C, D). The enrichment of phosphopeptides using TiO_2_ columns only led to the isolation of mono- and diphosphorylated peptide 1–21 (tryptic digest) and 1–20 (Glu-C digest, data not shown).

**Figure 4:**
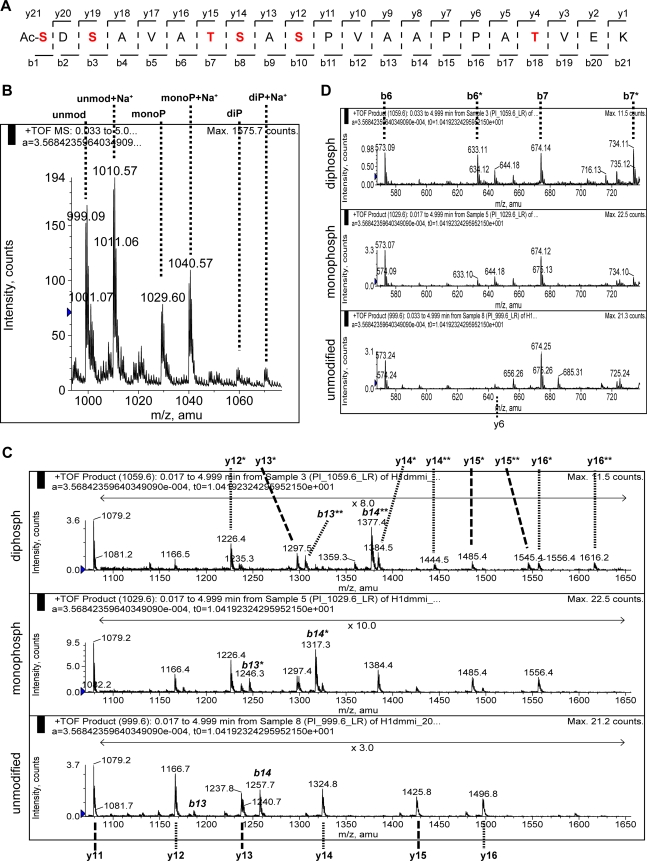
S10 is the major phosphorylation site in H1 isolated from 0–12 h embryos. H1 from 0–12 h embryos was purified, digested with trypsin, and submited to β-elimination followed by 1,4-addition of 2-mercaptoethanol. The resulting crude was desalted and the different forms of the peptide 1–21 were sequenced by ESI-MS/MS. A) Scheme showing the peptide 1–21 and the numbering of the b and y ions. The residues susceptible of phosphorylation are highlighted in red. B) TOF-MS spectrum of the sample. The double charged ions corresponding to 1–21, its modified forms and sodium adducts are indicated. Note that, due to the chemical treatment of the sample, the mass difference between the unmodified and the monomodified form is 60 instead of 80. C, D) Zooms of the product ion spectra for the unmodified (lower), monomodified (middle) and dimodified (upper) forms. * labels the ions with one modification; ** labels the ions with two modifications. C) For y11 only no modified ions are detected in both the mono- and dimodified peptide 1–21, which indicates that there is no detectable phosphorylation at T18. Therefore, the presence of monomodified y12 can only be due to phosphorylation at S10. The pattern of the b13 and b14 ions in the spectra of the mono- and dimodified species corroborate this assumption. In the spectrum of the diphosphorylated population, the presence of only monomodified y12 and y13 indicates that one of the two phosphate groups on each molecule is at S10. Additionally, the signal of dimodified y14 reveals the existence of a subpopulation of the peptide 1–21 containing phosphate groups at S10 and S8 simultaneously. Moreover, the higher relative intensity of dimodified vs. monomodified ions y15 and y16 respect to the same ratio for y14 suggests the presence of a species with simultaneous S10 and T7 phosphorylation. D) There is a small proportion of H1monophosphorylated at S1 and/or S3, as revealed by the intensities of b6, b6*, b7 andb7*. In the diphosphorylated population there is also modification at S1 and/or S3, however, simultaneous phosphorylation at S1 and S3 in the same molecule cannot be detected.

Because the peptide 1–21 has 6 residues (4 Ser and 2 Thr, Fig. 4A) that could be phosphorylated, we analyzed the mono- and diphosphorylated forms by ESI-MS/MS. H1 was extracted from 0–12 h embryos and digested with trypsin. The attempts to sequence 1–21 directly from this sample were unsuccessful due to the instability of the phosphate groups in the measurement conditions. To overcome this problem, the product of the trypsin digest was treated with barium hydroxide to eliminate the phosphate groups and subsequently submitted to a 1,4-addition with 2-mercaptoethanol (Fig 4). The increased stability of the modified peptides allowed mapping of the phosphorylation site by tandem mass spectrometry. In the TOF-MS spectrum only unmodified, mono- and diphosphorylated 1–21 can be observed, together with their sodium adducts (Fig. 4B). As we have discussed earlier, the analysis of all these species proves that the acetylation observed in 1–52 (see Fig. 1D) resides in the amino acids 1–3 and, given the nature of the first 3 aminoacids (SDS) and the fact that the acetylation is not seen in a peptide covering aminoacids 2–52 (Fig. 1B), one can firmly conclude that the N-terminus of the protein is acetylated.

Judging from the MS/MS spectra of the differentially modified peptides, we conclude that there is no detectable phosphorylation outside amino acids 1–10 and that most of the phosphorylation is found at position S10 in the singly- as well as the doubly phosphorylated peptide (see Fig. 4C). However, there also seems to be some minor subpopulations in the monophosphorylated H1 that carry the phosphate group at S1 or S3 (see y ions in Fig. 4C and b ions in Fig. 4D). Unfortunately, the absence of b1 and b2 ions makes it impossible to discriminate between S1 and S3 phosphorylation. We can therefore not distinguish if the monomodified b6 ion comes from a subpopulation of H1 monophosphorylated at S1 or from H1 monophosphorylated at S3 or if both subpopulations are present in the sample. The use of different collision energy values during acquisition did not improve the results. The same is true in case of the phosphorylation at T7 and/or S8 in the diphosphorylated peptide (Fig. 4C). The y ions indicate that there is some extent of phosphorylation at S8 and perhaps as well at T7, but the intensity of these ions and the corresponding b ions is too low to make any strong conclusion about the status of T7.

In summary at least two subpopulations of diphosphorylated H1 can be distinguished:

i) one of the phosphate groups is at S10 and the other at S1/S3; However, as mentioned above, we could not distinguish between S1 and S3 modification, but from the b ions (b6, b7 in Fig. 4D) it is clear that at least one of these two combinations (i.e., diphosphorylation at S10 and S1 or diphosphorylation at S10 and S3) is present in our sample.
ii) one of the phosphate groups is at S10 and the other at T7/S8. The presence of a dimodified y14 together with the absence of dimodified y12 and y13 indicates the existence of a phosphate group at S8. The apparent higher dimodified vs. monomodified ratio of y15 and y16 when compared to y14 suggests that there is also part of the population that is diphosphorylated at S10 and T7.

The analysis of the sodium adducts of the mono- and diphosphorylated forms yield the same result.

### Analysis of H1 phosphorylation in staged embryos

Once known that H1 in *D. melanogaster* embryos is phosphorylated at different positions in the first 10 amino acids, we addressed whether the proportion of phosphorylated protein and/or the positions that are modified change during embryonic development. We extracted H1 from embryos 2–3 h, 3–6 h, 6–9 h, 9–12 h and 12–15 h a.e.l., and analyzed the samples the same way as H1 from 0–12 h embryos. We have not analysed 0–2 h embryos because the synthesis of H1 begins with the cellularization i.e., approximately 2 h after egg laying. Indeed, in all our preparations, we got notably less H1 from the 2–3 h embryos than from the rest of the stages, even though the nuclear pellets were similar in size.

**Figure 5:**
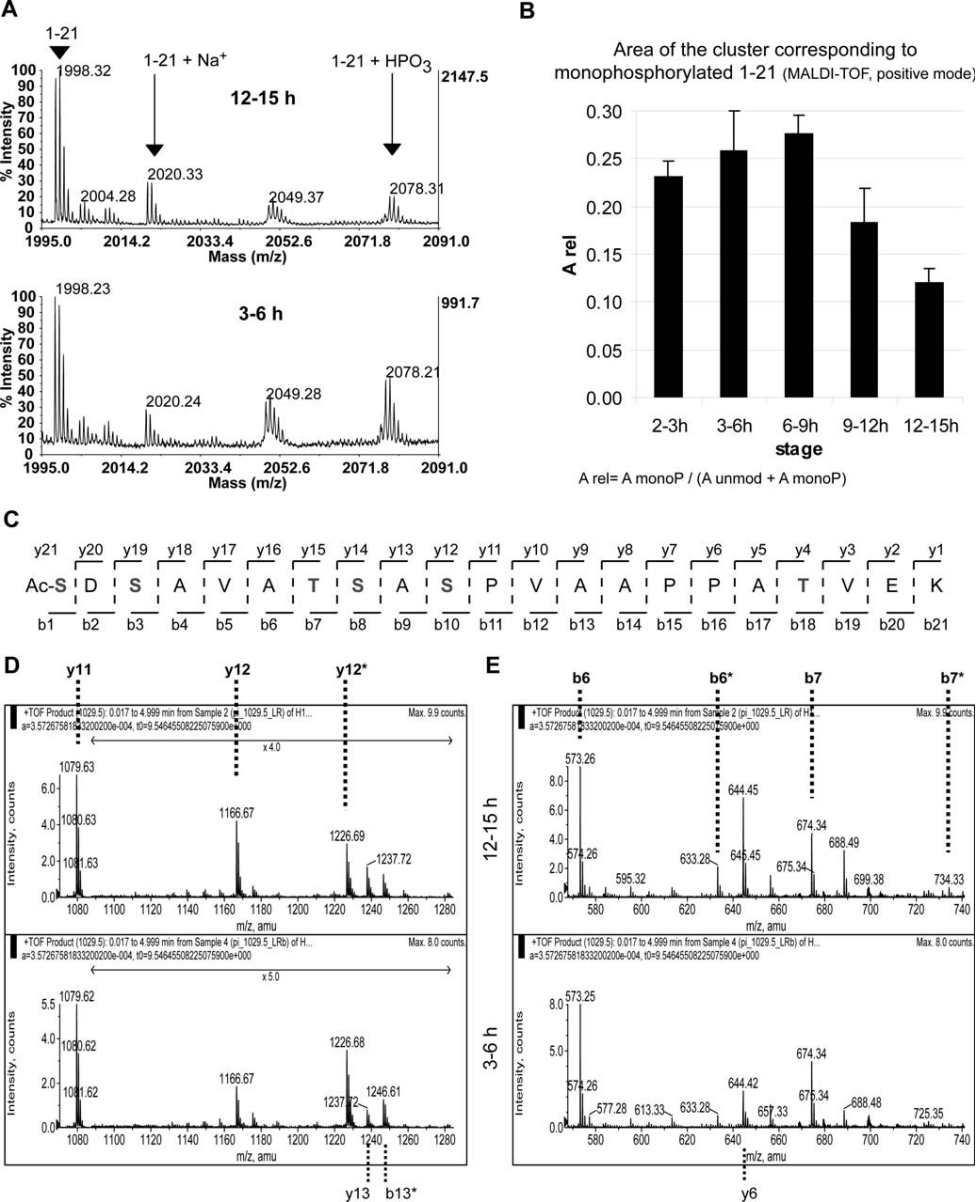
The monophosphorylation at S10 decreases as the embryos maturate. Histone H1 from 2–3 h, 3–6 h, 6–9 h, 9–12 h and 12–15 h was isolated and digested with trypsin. A) Zoom of two MALDI-TOF spectra acquired in the reflector positive mode are shown as examples. The upper spectrum corresponds to the 12–15 h old embryos, the lower to the 3–6 h ones. Note the different intensity of the signal at 2078. B) To compare the proportion of monophosphorylated H1 between the different stages, the area of the clusters corresponding to unmodified 1–21 (both proton and sodium adducts) and the monophosphorylated form (only proton adduct, sodium adduct not detected) was calculated for H1 from staged embryos. For each sample, the relative area of the monophosphorylated species (A_rel_) was calculated by division of the area of the monophosphorylated species through the total area (unmodified+monophosphorylated). The results of two independent biological replicates are shown. The error bars indicate the maximum and the minimum obtained values. C) Scheme showing the peptide 1–21 and the numbering of the b and y ions. The residues susceptible of phosphorylation are highlighted in red. D and E) H1 from 3–6 h and 12–15 h old embryos was purified, digested with trypsin, submitted to chemical modification (β-elimination and Michael addition) and finally, the forms of 1–21 were sequenced by ESI-MS/MS. Zooms of the product ions of the monomodified species are shown. * labels the modified species. D) The relative intensities of y12 and y12* indicate that the 12–15 h old embryos have less proportion of S10 phosphorylation than the younger embryos. E) The relative intensities of the b and b* ions suggest that monophosphorylated H1 from 12–15 h old embryos contains more proportion of S1 and/or S3 phosphorylation than the sample from the younger embryos.

The relative intensities of the MALDI-TOF spectra indicate a decrease in the proportion of monophosphorylated protein in the oldest embryos, independently of the protease (AspN or trypsin) used and the mode of spectra acquisition (linear or reflector, positive or negative). An example is shown in Fig. 5A. Integration of the area of the signals for unmodified (proton and sodium adducts) and monophosphorylated 1–21 clearly points out the loss of modified H1 (Fig. 5B). Due to the low intensity of the signals, it was not possible to conclude if there were also changes in the proportion of diphosphorylated peptide.

To determine if the loss of phosphorylation occurs selectively at a certain position, we determined the site of modification by ESI-MS/MS 3–6 h and 12–15 h embryos. The product ion spectra of the ion containing the monophosphorylated H1 peptide in both samples show noticeable changes in the relative intensities of unmodified vs. modified y12 (Fig. 5D) demonstrating that the major site of monophosphorylation in the younger embryos is S10, whereas in the older embryos the proportion of monophosphorylation occurring at S10 is smaller. Besides the main phosphorylation site at S10 we also find a smaller fraction of the monophosphorylated peptide to be modified at position S1 or S3 as indicated by the existence of an unmodified y12 ion (y12 Fig 5D) and a modified b6 ion ( b6*, Fig 5E). Interestingly the relative intensity of the modified versus unmodified b6 ion is higher in older embryos (b6*/b6 = 0.21±0.02) compare to younger ones (b6*/b6 = 0.13±0.03) suggesting that the decrease in monophosphorylation is due to a decrease in S10 phosphorylation whereas the phosphorylation at the N-terminus remains unchanged.

In summary, during the first hours of development, the proportion of H1 phosphorylated at S10 is decreasing.

## Discussion

We present here a detailed analysis of PTMs in H1 from *D. melanogaster* embryos using mass spectrometry. The results show that the single most prominent PTM in these samples is a phosphorylation at S10. Other groups had already reported that phosphorylation in *D. melanogaster* H1 is found in the N-terminal region of the protein, but the exact amino acids, which carry the modification remained unidentified [36]. There are several interesting features that distinguish H1 phosphorylation in *Drosophila* from H1 phosphorylation in mammals. First, in mammalian cells, H1 variants with 4, 5 or even 6 phosphate groups are detected [20], [37], whereas in *Drosophila* species we and others only detect mono- [36], [38] and diphosphorylated H1 [39]. Second, many mammalian H1 proteins have phosphorylation sites are in the C-terminus, which are often found to be consensus CDK-phosphorylation sites [20], [26]. In contrast, *D. melanogaster* H1 lacks these (S/T)PXZ (where X is any amino acid and Z a basic amino acid) sequences and no phosphorylation at the C-terminal region has been detected so far. Because in mammalian cells the levels of H1 phosphorylation increase as the cell cycle progresses from G1 to mitosis, it has been suggested that CDK kinases are responsible for the phosphorylation of H1. However, research performed in different laboratories has shown that, at least in mammalian H1, the mitosis specific phosphorylation occurs in the N-tail of the protein, in sites that do not fit with the (S/T)PXZ sequence [20], [37]. On the one hand, in chinese hamster ovary cells, the mitosis specific phosphorylation is found in S1 and T3 [37], on the other hand, in human H1.5, it is found in T10. Despite a general lack of interspecies homology within the tail regions of H1 molecules, the extreme N-terminus of most H1 molecules is reasonably well conserved. Based on this similarity and on the observations discussed above we suggest that the phosphorylation we observe is mitosis dependent. This would also explain the reduction of the proportion of monophosphorylated H1 as the embryos get older and the number of mitotically active cells decreases.

Together with phosphorylation, lysine acetylation, methylation and formylation have been found in mammalian H1, but very little about their biological function is known [19], [24]–[27], [40]. In our study we have not detected any PTM besides phosphorylation. Two reasons may explain this fact: First, we have employed a different model organism, in which H1 is present as a single variant and which already differs with the other organisms in the position of the major phosphorylation sites. Second, we cannot rule out the existence of a minor proportion of H1 carrying any of these PTMs in *Drosophila*, whose detection would require the use of different analytical techniques or additional enrichment steps.

Finally, we would like to point out that the existence of polymorphisms in the protein sequence can lead to erroneous interpretations. Diverse couples of amino acids have a similar chemical structure that differs only in the presence of a methyl group. This is the case for glycine and alanine, alanine and valine, valine and leucine or isoleucine, aspartic and glutamic acid, asparagine and glutamine, serine and threonine. Thus, if the peptides are not fully sequenced with all fragment ions visible, it is not possible to assess if the observed 14 units mass difference is due to a methyl group modifying a lysine or arginine residue or to a polymorphism in the protein sequence. Additionally, the mass difference between lysine and arginine is very similar to the mass difference between an unmodified and dimethylated lysine and can only be distinguished with mass spectrometers of very high resolution. Existence of these kind of substitutions in human H1.2 and H1.4 have already been reported [20].

In summary, we have analyzed the entire sequence of *D. melanogaster* H1 by mass spectrometry. Apart from the N-terminal acetylation, we have only found mono- and diphosphorylation in the first 10 aminoacids. The major site of phosphorylation is S10, however minor phosphorylation sites can also be found at S1 or 3 and at T7 or S8. The proportion of monophosphorylated H1 decreases as the embryos mature, mostly due to a decrease in the phosphorylation at S10. This fact together with previous findings about mammalian H1, suggest that this modification is regulated during mitosis.

## Materials and Methods

### Histone H1 isolation from embryos

Embryos were washed from the agar plates with tap water and dechorionated by stirring for 1 min in diluted bleach solution (23 ml bleach 6–14% active chlorine in 100 ml final volume), rinsed with 1 l 0.12 M NaCl containing 0.04% Triton X-100 (v/v) and washed thoroughly with tap water. 4–5 g dechorionated embryos were dehydrated by immersion in 70% ethanol during 5 min at room temperature, decanted and stored at −80°C.

Nuclei from the frozen embryos were prepared in a similar way as described before [38]. Briefly, 4–5 g frozen embryos were thawed at 4°C in 8–10 ml buffer 1 (0.35 M sucrose, 15 mM HEPES pH = 7.6, 10 mM KCl, 5 mM MgCl_2_, 0.5 mM EGTA pH = 8.0, 0.1 mM EDTA pH = 8.0, 10 mM glycerolphosphate, 10 mM sodium butyrate, 10 mM NaF, 1 mM DTT, 0.2 mM PMSF, 1 mM Na_2_S_2_O_5_). The suspension was passed twice through a Yamato LH-31 homogenizer at 1000 rpm and the homogenate was filtered through a layer of Miracloth. Filtrates were transferred to 50 ml tubes, diluted with buffer 1 up to 5 ml/g embryo and centrifuged (3000 g, 4°C, 20 min). Supernatants were decanted and the nuclear pellet was washed twice with buffer 1 supplemented with 0.1% NP-40 (wash buffer), first employing 5 ml wash buffer/g embryo and next with 2 ml wash buffer/g embryo.

Nuclei were transferred to 1.5 ml tubes and extracted with diluted HClO_4_ as described previously [21], [41], [42]. Briefly, nuclei were resuspended in 3 vols 6.7% HClO_4_ and rotated at 4°C 1 h. After centrifugation (16000 g, 5 min, 4°C), supernatants were kept in clean tubes whereas pellets were reextracted with 1 ml 5% HClO_4_. The HClO_4_ extracts were dialysed against 0.1 M acetic acid containing 1 mM DTT (membrane MWCO 6000–8000, 2×0.5 l, 1×1l), lyophilyzed, reconstituted in 20 mM Tris pH = 6.8, 1mM DTT and stored at −20°C. The quality of the preparation was checked by SDS-PAGE. In none of the second HClO_4 _extracts H1 could be detected.

### Histone H1 isolation from SL2 cells

SL2 cells were grown at 26°C in Schneideŕs *Drosophila* medium with L-glutamine supplemented with 10% FBS, 100 U/ml penicilin and 100 µg/ml streptomycin. Cells were harvested by centrifugation (500 g, 10 min, room temperature) and directly resuspended in 3 vols 6.6 % HClO_4_. The rest of the procedure is identical to that one employed for the embryos.

### Histone H1 purification

H1 was purified by HPLC over a Jupiter C4 (nucleosil 5 µm, 300 Å 150×1 mm) column (Phenomenex) with 0.05% TFA as eluent A and 0.065% TFA/84% acetonitrile as eluent B. Elution was performed with a 3-steps gradient (31%B during 2 min, 31%B to 37% B in 10 min, 37% B to 45% B in 30 min) at 50 µl/min flow rate and absorbance at 214 nm was monitored. 50 µl fractions were collected. Before proceeding to the next sample, column was washed with 100% B during 10 min and reequilibrated with 31% B. For each run, fractions containing H1 were pooled, concentrated to dryness (speed-vac), reconstituted in 10–30 µl 0.1% TFA and stored at −20°C.

### Histone H1 protease digestion

HPLC-purified H1 (amount corresponding to 30 mAU/min in the chromatogram) was digested in a reaction volume of 20 µl overnight at 25°C with 80 ng AspN (Roche) in 0.1 M Tris pH = 8.5; or at 25°C with 50 ng GluC (Roche) in 25 mM NH_4_HCO_3_ or at 37°C with 0.4 µg trypsin (Promega) in 50 mM NH_4_HCO_3_. Except otherwise stated, reaction crudes were quenched with 2 µl TFA.

### MALDI-TOF MS analysis

3 µl each quenched digestion crude were diluted with 7 µl 0.1% TFA and desalted with ZipTip µ-C18 (Millipore) according manufactureŕs instructions. Samples were eluted from the resin with 2 µl 50% acetonitrile/0.1% TFA, mixed with an identical volume of matrix (50% acetonitrile/0.3% TFA, saturated of α-cyano-hydroxycinnamic acid) and spotted immediately onto a stainless steel MALDI-TOF target plate. Spectra were acquired on a Voyager DE STR workstation (Applied Biosystems).

Integration of the signals corresponding to each peptide (isotopic cluster area) was automatically performed by the Data Explorer software (Applied Biosystems) excluding all the peaks whose signal-to-noise ratio was smaller than 4.

### Carboxypeptidase Y analysis of the peptide 189–255

Quenched AspN digestions were pooled to get approximately 300 µl final volume and concentrated to ca. 80 µl. Formic acid was added (final concentration: 0.1%) and 40 µl were fractionated on a Gemini C18 column run at 0.2 ml/min. Eluent A was 0.05% TFA and eluent B 0.065% TFA/84 % acetonitrile; gradient from 4% B to 25% B in 60 min. 0.1 ml fractions were collected. Absorbance at 214 nm was monitored and 2 µl each fraction were used to analyze its composition by MALDI-TOF MS in positive, linear mode.

Fractions corresponding to the unmethylated and monomethylated forms of 189–255 were collected, concentrated to dryness and reconstituted in 1 µl reaction mix (50 mM Tris pH = 6.8 and 40 ng/µl carboxypeptidase Y). 5 µl each reaction were quenched with 5 µl 2.5 % TFA after 0.5 h incubation and the rest, after 2.5 h. Quenched digestion mixtures were directly desalted and analyzed by MALDI-TOF as indicated above.

### Beta-elimination and Michael addition

For each sample, 1-2 tryptic digests (not quenched) were pooled and divided in 5 µl aliquots. Chemical modification was performed as described by Arrigoni et al. [43] with some modifications. Each aliquot was mixed with 20 µl 0.1 M Ba(OH)_2_ and incubated at 50°C for 30 min. Immediately 2.6 µl 0.1 M beta-mercaptoethanol were added to each crude and the mixtures were kept at 50°C for further 30 min. Each aliquot was quenched with 5 µl 10% TFA, aliquots were pooled and desalted with Perfect Pure C18 Tips (Eppendorf) according manufactureŕs instructions, replacing TFA for formic acid in all the buffers. Samples were eluted in 3–10 µl 50% MeOH/0.1% formic acid and analysed by ESI-MS/MS.

### ESI-MS/MS analysis

Tandem mass spectrometry analyses were performed on a Qstar XL hybrid quadrupole TOF spectrometer (Applied Biosystems). For sample ionization, either a nanospray ion source (Protana) or a Nanomate (Advion) were employed. For each spectrum, measurements during 5 min were accumulated. Acquisition was performed in the positive mode and the collision energy was manually adjusted during the measurements, using the same values during the same time for all the samples.

Spectra were manually interpreted.

### DNA extraction

Nuclei from 0.6 g *D. melanogaster* dechorionated embryos were prepared as described above. Nuclei from SL2 cells were prepared as follows: about 50×10^6^ cells were harvested (centrifugation at 500 g during 10 min at RT) and resuspended in 1.0 ml RSB (10 mM Tris pH = 7.5, 10 mM NaCl, 3 mM MgCl_2_). 0.1 ml 10% NP-40 was added and the suspension was incubated on ice during 10 min. After centrifugation (2000 g, 4°C, 5 min) the nuclear pellet was washed once with 1 ml RSB.

Nuclei from SL2 cells or from embryos were lysated in 0.5 ml proteinase K buffer (50 mM Tris pH = 8.5, 10 mM EDTA pH = 8.0, 0.1 M NaCl and 1% SDS). 20 µg RNAse A were added to each sample, followed by 30 min incubation at 30°C. Then, 40 µg proteinase K were added and digestion was allowed to proceed at 37°C overnight. After a wash with 1 vol phenol (saturated with TE) and another wash with phenol/chloroform/isoamylalcohol (50/49/1), DNA was precipitated with 1.5 ml absolute ethanol in the presence of 50 µl 3 M sodium acetate. DNA was pelleted (centrifugation at 16000 g, 2 min, r.t.), washed twice with 70% ethanol, reconstituted in water and stored at −20°C.

### PCR

0.1 µg genomic DNA was amplified with T4 Taq Polymerase (NEB) according manufactureŕs instructions in a 50 µl reaction containing 1.0 µM each primer (forward: GGATGCCAAGAAAACTGGA, reverse: TTTTTGGCAGCCGTAGTCTT). 35 cycles were performed with 60°C annealing temperature. The Quality of the product was checked by agarose gel electrophoresis with ethidium bromide staining.

### Restriction enzyme digestion

0.5 µg PCR product (purified with the Illumina GFX PCR DNA and gel band purification kit, Amersham, according manufactureŕs instructions) were digested with 5 U SfcI (NEB) in 50 µl reaction, according manufactureŕs instructions. To reach the complete digestion, after 1.5 h incubation at 37°C, 5 U SfcI more were added to each reaction and the incubation was prolonged overnight at 30°C. Reaction products were analyzed on an 8% acrylamide gel with ethidium bromide staining.
